# Supplementary material for: Polygenic transcriptome risk scores enhance predictive accuracy in atopic dermatitis
Source: J Transl Med. 2025 May 23;23:575. doi: 10.1186/s12967-025-06570-8 (PMC12102998; doi:10.1186/s12967-025-06570-8)

**Polygenic transcriptome risk scores enhance the predictive accuracy in atopic dermatitis**

Charalabos Antonatos, Ashley Budu-Aggrey, Lavinia Paternoster, Alexandros Pontikas, Adam Akritidis, Sophia Georgiou, Aikaterini Tsiogka, Stamatis Gregoriou, Katerina Grafanaki, Yiannis Vasilopoulos

**Supplementary Figures**

Fig. S1. High-resolution PRSicev2 plot reporting the predictive accuracy of PRS across various P-value thresholds in the training dataset.

Fig. S2. Upset plot showing the number of overlapping genes included in each best performing, tissue-specific PTRS model.

Fig. S3. Biological processes involved in genes mapped across tissue-specific, best performing PTRS models.

Fig. S4. Pearson correlation estimates between standardized PRS and standardized PTRS in EBV transformed lymphocytes.

Fig. S5. Pearson correlation estimates between standardized PRS and standardized PTRS in lung.

Fig. S6. Pearson correlation estimates between standardized PRS and standardized PTRS in not sun exposed skin.

Fig. S7. Pearson correlation estimates between standardized PRS and standardized PTRS in sun exposed skin.

Fig. S8. Pearson correlation estimates between standardized PRS and standardized PTRS in small intestine.

Fig. S9. Pearson correlation estimates between standardized PRS and standardized PTRS in spleen.

Fig. S10. Pearson correlation estimates between standardized PRS and standardized PTRS in whole blood.


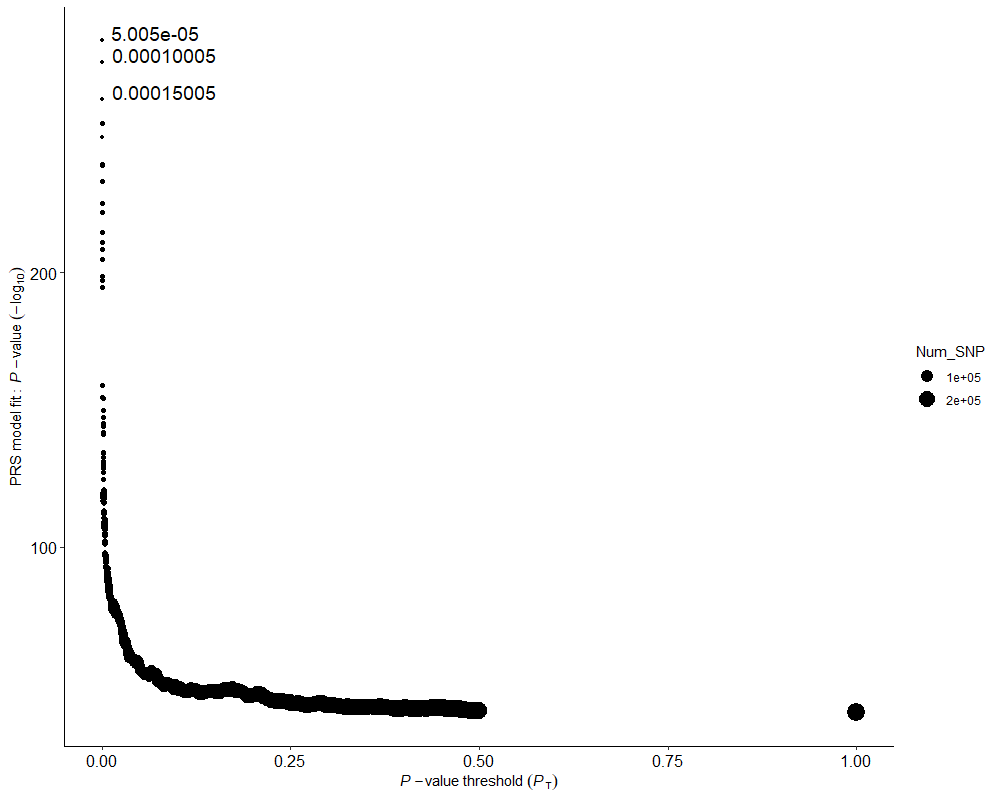
Fig. S1. High-resolution PRSicev2 plot reporting the predictive accuracy of PRS across various P-value thresholds in the training dataset.

Fig. S2. Upset plot showing the number of overlapping genes included in each best performing, tissue-specific PTRS model.


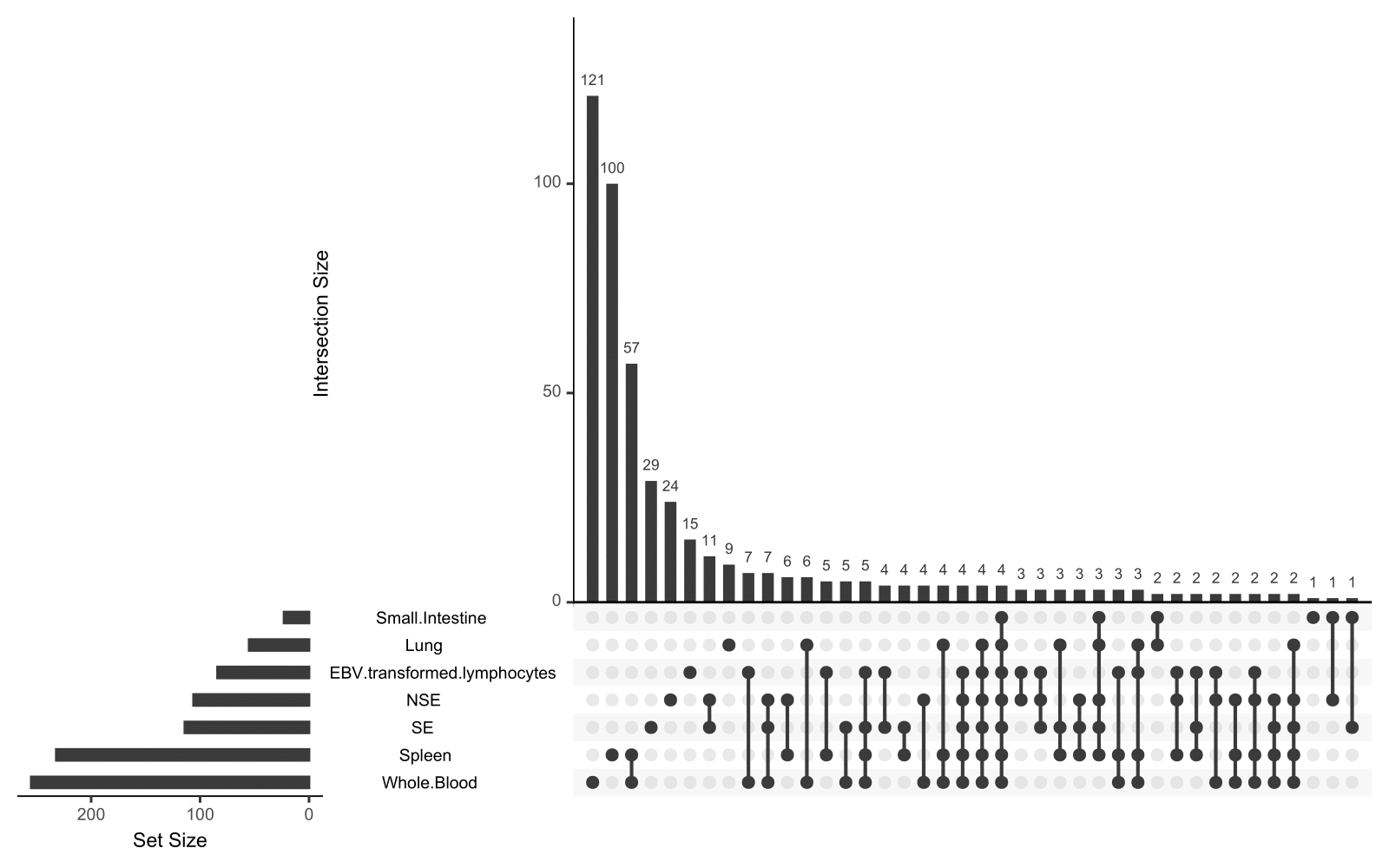


Fig. S3. Biological processes involved in genes mapped across tissue-specific, best performing PTRS models.
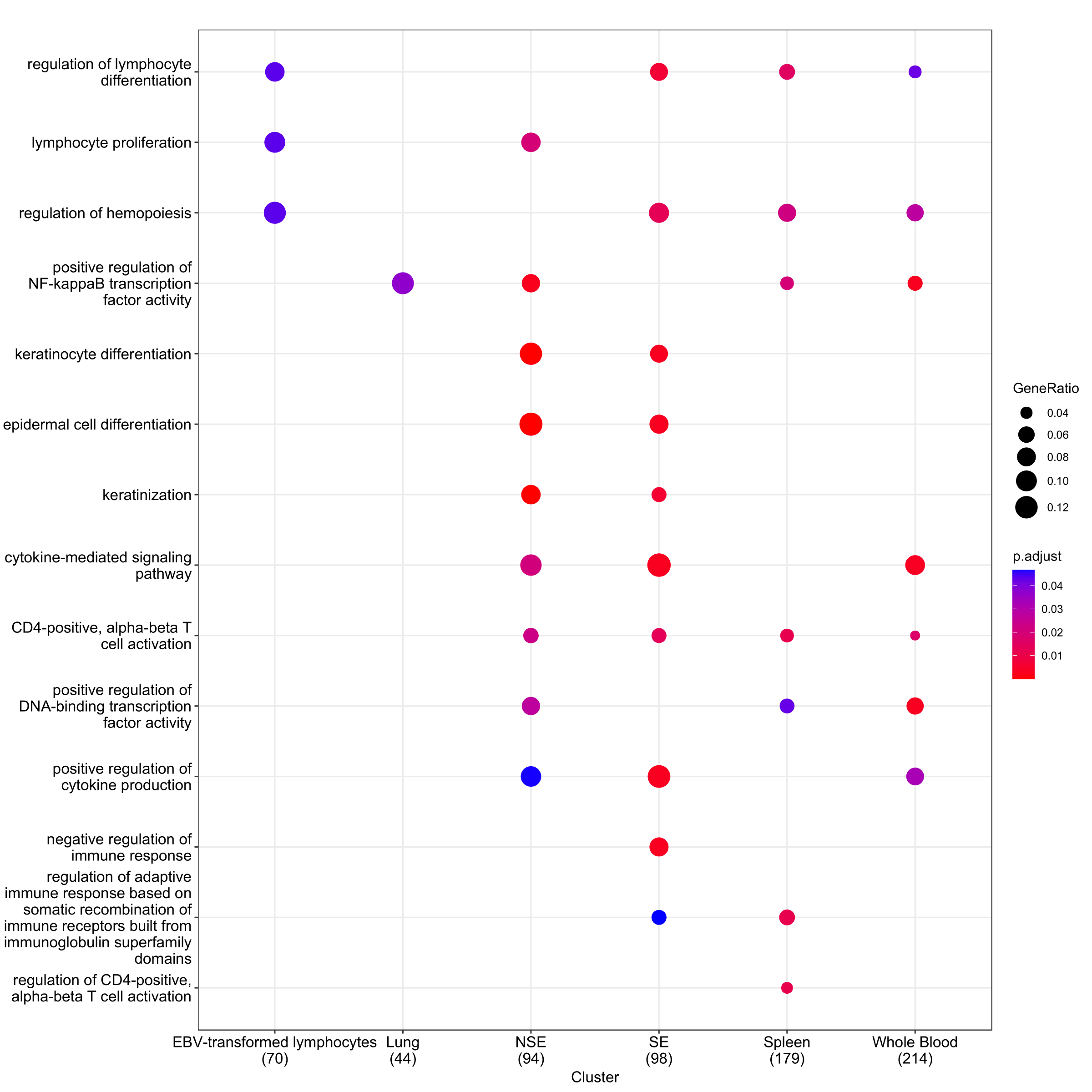


Fig. S4. Pearson correlation estimates between standardized PRS and standardized PTRS in EBV transformed lymphocytes.


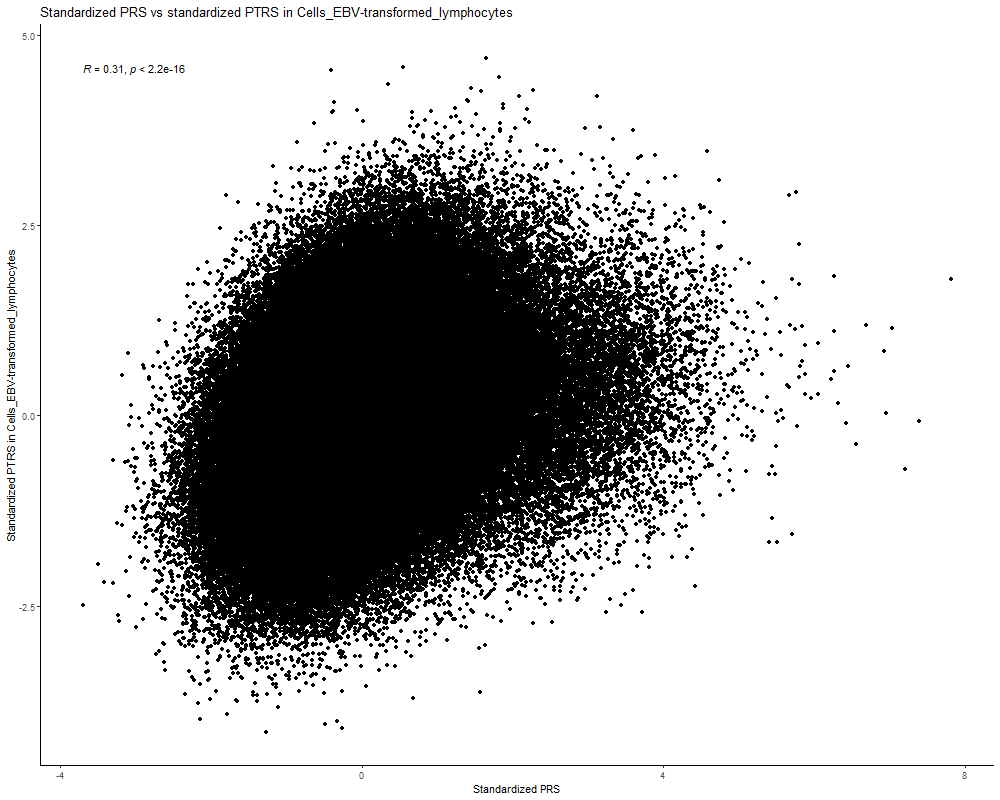


Fig. S5. Pearson correlation estimates between standardized PRS and standardized PTRS in lung.


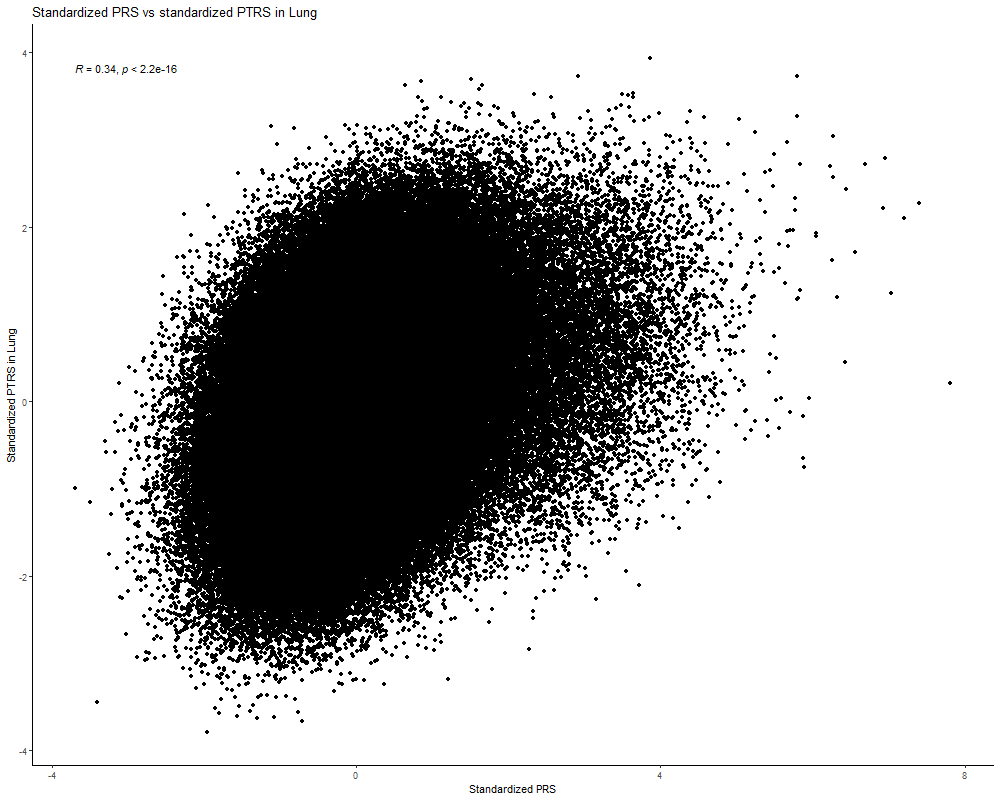


Fig. S6. Pearson correlation estimates between standardized PRS and standardized PTRS in not sun exposed skin.


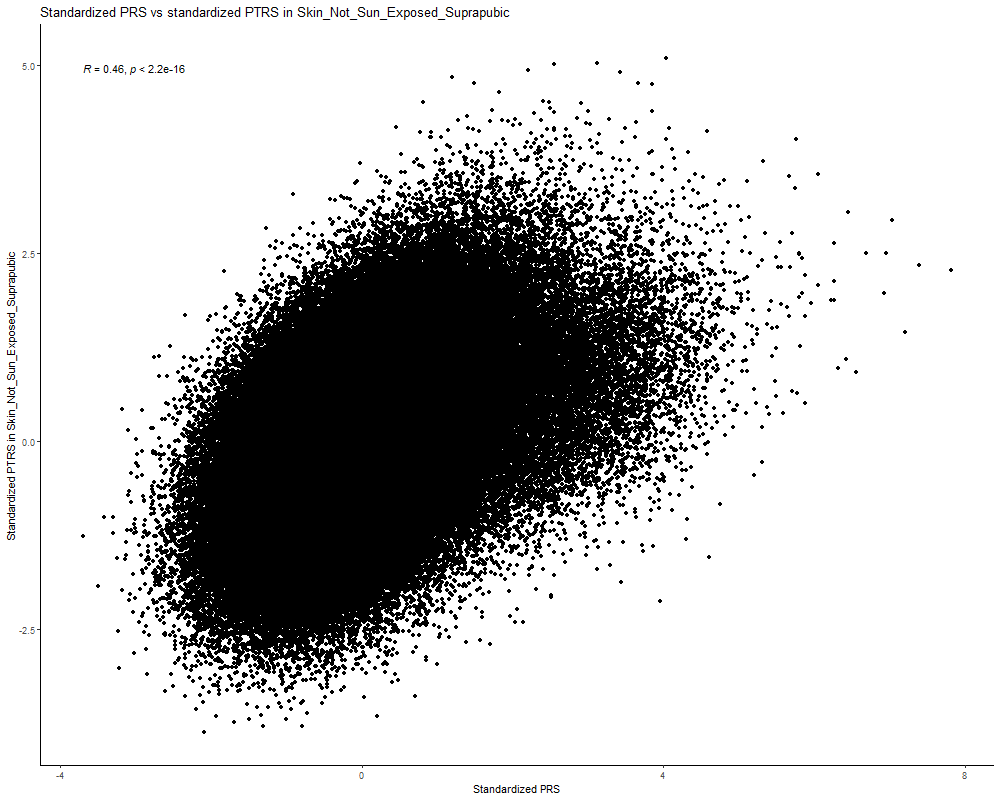


Fig. S7. Pearson correlation estimates between standardized PRS and standardized PTRS in sun exposed skin.


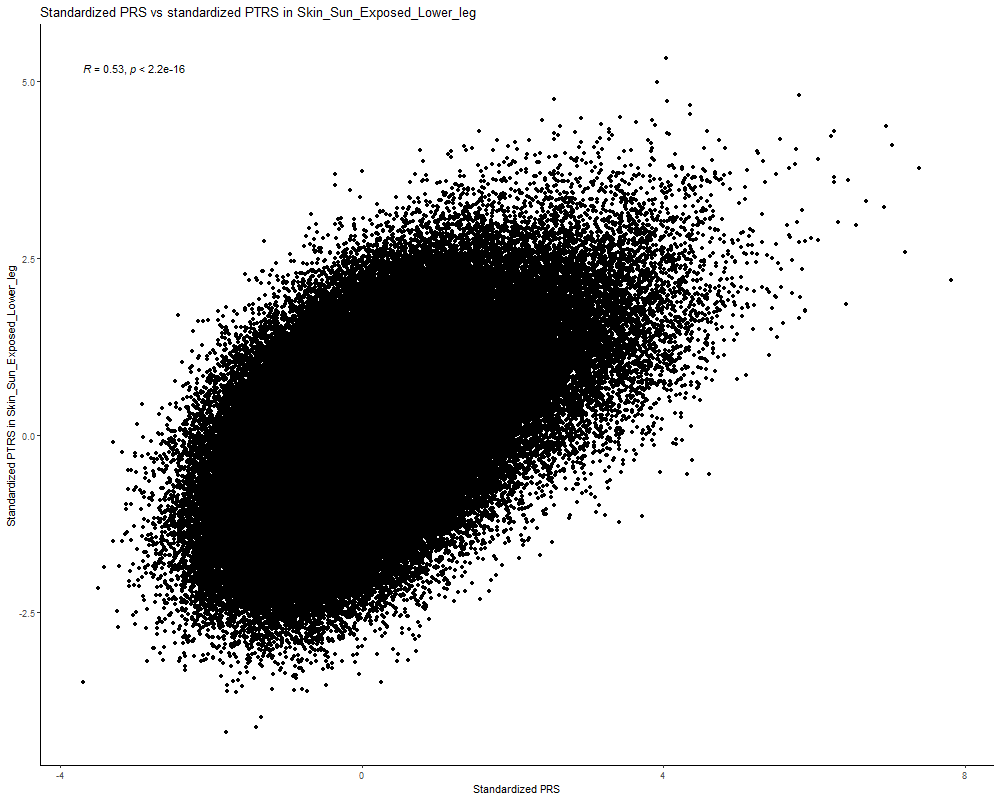


Fig. S8. Pearson correlation estimates between standardized PRS and standardized PTRS in small intestine.


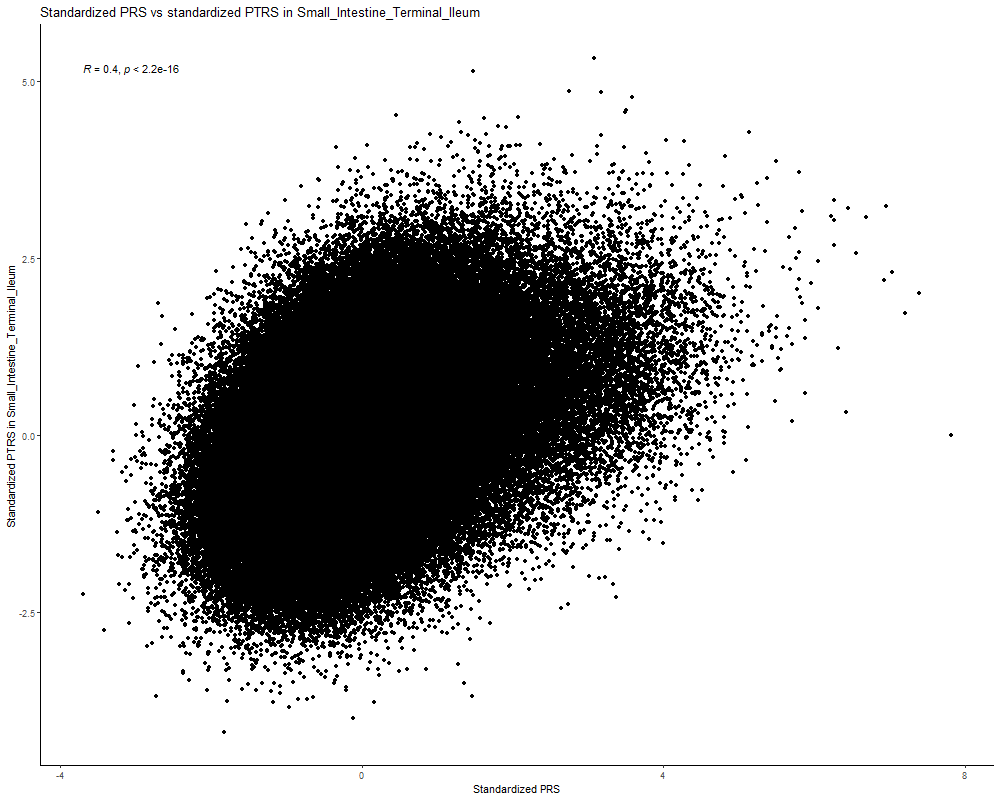


Fig. S9. Pearson correlation estimates between standardized PRS and standardized PTRS in spleen.


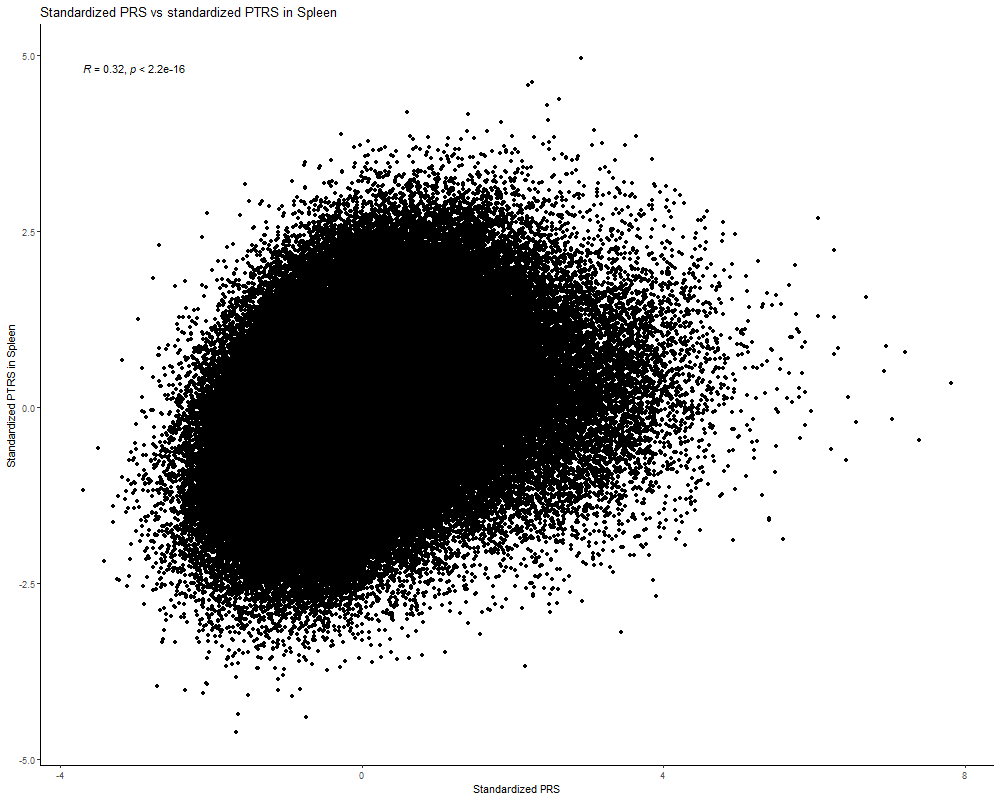


Fig. S10. Pearson correlation estimates between standardized PRS and standardized PTRS in whole blood.


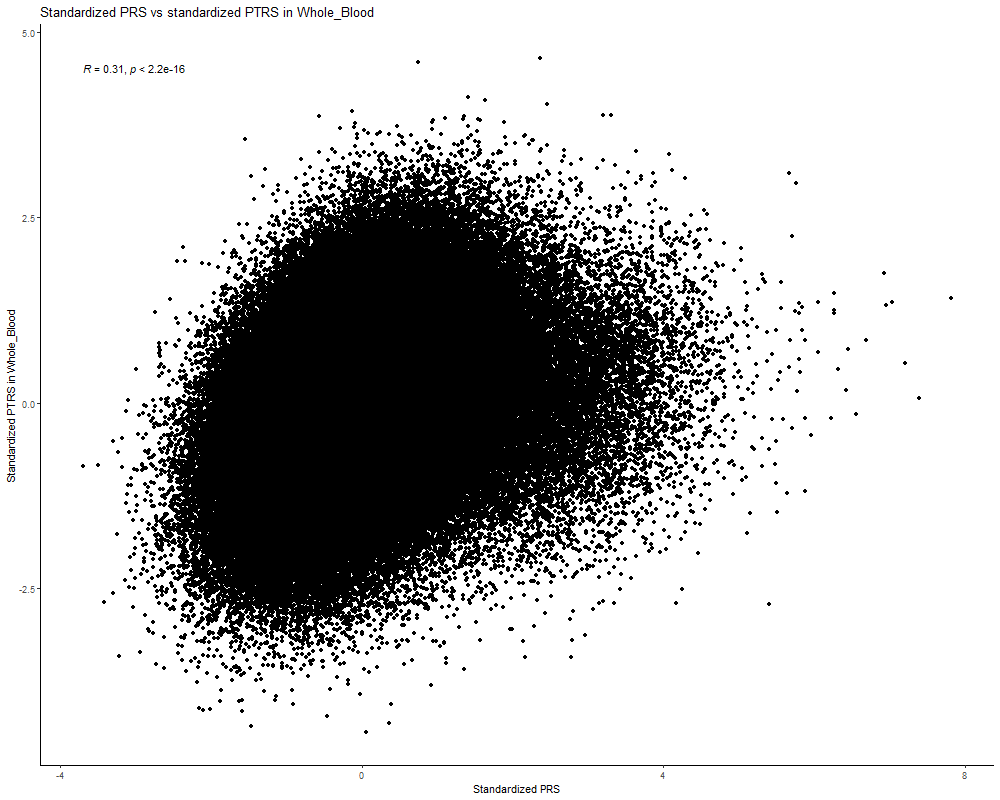

Supplement: Supplementary file 2 — Supplementary materials 2: Fig. S1. High-resolution PRSicev2 plot reporting the predictive accuracy of PRS across various P-value thresholds in the training dataset. Fig. S2. Upset plot showing the number of overlapping genes included in each best performing, tissue-specific PTRS model. Fig. S3. Biological processes involved in genes mapped across tissue-specific, best performing PTRS models. Fig. S4. Pearson correlation estimates between standardized PRS and standardized PTRS in EBV transformed lymphocytes. Fig. S5. Pearson correlation estimates between standardized PRS and standardized PTRS in lung. Fig. S6. Pearson correlation estimates between standardized PRS and standardized PTRS in not sun exposed skin. Fig. S7. Pearson correlation estimates between standardized PRS and standardized PTRS in sun exposed skin. Fig. S8. Pearson correlation estimates between standardized PRS and standardized PTRS in small intestine. Fig. S9. Pearson correlation estimates between standardized PRS and standardized PTRS in spleen. Fig. S10. Pearson correlation estimates between standardized PRS and standardized PTRS in whole blood. [file 12967_2025_6570_MOESM2_ESM.docx]
